# Family health climate scale (FHC-scale): development and validation

**DOI:** 10.1186/1479-5868-11-30

**Published:** 2014-03-05

**Authors:** Christina Niermann, Fabian Krapf, Britta Renner, Miriam Reiner, Alexander Woll

**Affiliations:** 1 Department of Sports Science, University of Konstanz, P.O. Box 30, D-78457 Konstanz, Germany; 2 Department of Psychology, Psychological Assessment and Health Psychology, University of Konstanz, P.O. Box 47, D-78457 Konstanz, Germany; 3 Karlsruhe Institute of Technology, Institute of Sports and Sports Science, Engler-Bunte-Ring 15, D-76131 Karlsruhe, Germany

**Keywords:** Health behaviour, Physical activity, Nutrition, Family environment, Scale development

## Abstract

**Background:**

The family environment is important for explaining individual health behaviour. While previous research mostly focused on influences among family members and dyadic interactions (parent**-**child), the purpose of this study was to develop a new measure, the *Family Health Climate Scale* (FHC-Scale), using a family-based approach. The FHC is an attribute of the whole family and describes an aspect of the family environment that is related to health and health behaviour. Specifically, a questionnaire measuring the FHC (a) for nutrition (FHC-NU) and (b) for activity behaviour (FHC-PA) was developed and validated.

**Methods:**

In Study 1 (N = 787) the FHC scales were refined and validated. The sample was randomly divided into two subsamples. With random sample I exploratory factor analyses were conducted and items were selected according to their psychometric quality. In a second step, confirmatory factor analyses were conducted using the random sample II. In Study 2 (N = 210 parental couples) the construct validity was tested by correlating the FHC to self-determined motivation of healthy eating and physical activity as well as the families’ food environment and joint physical activities.

**Results:**

Exploratory factor analyses with random sample I (Study 1) revealed a four (FHC-NU) and a three (FHC-PA) factor model. These models were cross-validated with random sample II and demonstrated an acceptable fit [FHC-PA: *χ*^2^ = 222.69, df = 74, p < .01; *χ*^2^/df = 3.01; CFI = .96; SRMR = .04; RMSEA = .07, CI .06/.08; FHC-NU: *χ*^2^ = 278.30, df = 113, p < .01, *χ*^2^/df = 2.46, CFI = .96; SRMR = .04; RMSEA = .06, CI .05/.07]. The perception of FHC correlated (p < .01) with the intrinsic motivation of healthy eating (r = .42) and physical activity (r = .56). Moreover, parental perceptions of FHC-NU correlated with household soft drink availability (r = -.31) and perceptions of FHC-PA with the frequency of joint physical activities with the child (r = .51). These patterns were found on the intraindividual and interindividual level.

**Conclusions:**

Two valid instruments measuring the FHC within families were developed. The use of different informants’ ratings demonstrated that the FHC is a family level variable. The results confirm the high relevance of the FHC for individuals’ health behaviour. The FHC and the measurement instruments are useful for examining health-related aspects of the family environment.

## Introduction

A person’s choice of the type of activity performed during leisure time (regular exercises or playing on the PC) or the type of food consumed every day (healthy foods or ‘empty calories’) is determined by the person’s cognition, emotion, motivation, and volition. However, according to Bandura’s Social-Cognitive Theory this behaviour can only be explained by also considering the interaction of personal, behavioural, and environmental factors [1]. The individual does not live in a vacuum: the individual’s behaviour, his or her motives, emotions and cognitive processes and his or her social and physical environment are interrelated. Therefore, the present study focuses on the family as an important social environmental dimension that shapes the individual’s health behaviour and has a lasting effect [2].

### Family as environmental factor

To date, family research has mostly focused on the influence among family members, especially on the interaction between dyads and the parent**-**child relationships [3,4]. This becomes particularly apparent in the context of health behaviour: extensive research on parental influences on children’s and adolescents’ behaviour has shown that parents play an important role in the development of a healthy lifestyle [5-8]. Important mechanisms regarding nutrition and activity behaviour include direct influences such as modelling, monitoring, support, and encouragement [9-18], and indirect influences through parenting styles or parent**-**child bonding [19-24] and via effects on attitudes, values, self-efficacy-beliefs, self-control abilities or self-esteem [25-27]. However, family influences regarding a healthy lifestyle are not limited to the parents influencing their children: the members of a family are interdependent and exert an enduring and reciprocal influence on each other [3]. Using a systems metaphor for understanding families, a family is more than the sum of its parts and has properties that do not only reflect, but even go beyond the added-up characteristics of the single family members. This approach is illustrated in the theoretical framework of the Model of Family Reciprocal Determinism [6,28]. A family is a group of individuals and all members of this group have specific motives, affects, and behaviours. The individuals within a family interact with and reciprocally influence each other. These interactions take place over an extended time period and with a high frequency and constitute a ‘family system’ representing an essential component of the family environment.

### Family climate

According to the family-as-system approach, the crucial questions are how the family environment influences the individual’s health behaviour and how this influence can be described. It is proposed that a specific aspect of the family members’ interrelationships shapes the individuals’ activity and eating behaviour, and this aspect has been termed ‘climate’.

The climate concept consists of an aggregation of collectively shared opinions, attitudes, feelings, and behaviours that characterize life in a social setting [29,30]. Climate can be considered as attribute of a specific social setting rather than that of single members of this setting [29,31]. However, the psychological climate is commonly used as a property of the individual [32]. Therefore, the climate can be analysed on the group level by aggregating individual scores [29,31] or the climate perceptions can be analysed on the individual level [32]. Both levels of analysis are equally appropriate for the climate concept but must be selected depending on the purpose of the study. Carr et al. [33] and Parker et al. [32] suggested that the collective level of analysis is appropriate for studying outcomes in the organizational, school, or family level and the individual level of analysis is appropriate for analysing individual behaviour, individual well-being, or individual performance.

Moreover, molar (organizational climate [30], school climate [34], family climate [35]) and specific climate (safety climate [36], organizational health climate [37]) concepts have been described in the literature. The spectrum of the outcomes determines if a molar or a specific climate construct should be applied where specific climates are predictive for specific outcomes [33]. Assuming climate as a determinant of health behaviour enables a close view on the climate construct. In the context of describing a family environmental dimension that shapes the individual health behaviour, the term climate is seen as a specific attribute of the family judged by the individual. Therefore, the term *Family Health Climate* is introduced.

### Family health climate (FHC)

We propose to define the Family Health Climate as the shared perceptions and cognitions concerning health and health behaviour. It reflects the individual experience of daily family life, the evaluation of health-related topics and expectations with respect to typical values, behaviour routines and interaction patterns within the family. The Family Health Climate serves as a framework for an individual’s daily health behaviour. It is the basis of regulating health-related behaviours and provides references for valuing and interpreting their own behaviour and that of others. Hence, the Family Health Climate is an aspect of the family environment that shapes the daily health behaviours of the family members, both within and outside of the family.

### Family health climate and health behaviour

The main purpose of the Family Health Climate construct is to analyse the climate as a determinant of individual health behaviour, such as physical activity and nutrition. The evaluation of the Family Health Climate reflects shared cognitions and perceptions concerning a healthy lifestyle within a family. A positive Family Health Climate reflects that both being physically active and eating healthy is a very important and integral part of a family’s daily life.

Therefore, the perception of the Family Health Climate should be associated with the individual’s cognitive, motivational, and behavioural variables, with interactions related to physical activity or nutrition within the family, and with routines in family life. It is assumed that valuing the Family Health Climate positively reflects that a healthy lifestyle is internalized, which implies that both regular physical activity and healthy eating habits are regulated highly autonomously. Therefore, positive correlations with intrinsic and identified motivation and a negative correlation with amotivation concerning the respective behaviour are assumed. Moreover, a positive perception of the Family Health Climate is expected to be associated with support between family members and with family meals and joint physical activities, too. Finally, healthy foods should frequently be available in the household whereas unhealthy foods should be less frequently available.

As the perception of the FHC is assumed to represent a family-level variable, it is necessary to take into account the individual perspective as well as the perspective of interrelated family members. This implies that the perception of the FHC of one family member should be related to cognitive, motivational, and behavioural variables of another family member.

The aims of this study were to develop a preliminary version of the Family Health Climate Scale (FHC-Scale) in successive steps (Pre-Study), to refine the FHC-Scale and to validate its factorial structure (Study 1), and to determine the scale’s construct validity (Study 2) (for an overview see Additional file 1). All presented substudies were conducted within the research project ‘EATMOTIVE’ funded by the Federal Ministry of Education and Research, Germany.

#### Pre-study: item generation and development of the family health climate scale (FHC-scale)

Because specific climate perceptions predict specific behaviours [33] and eating and exercising – although they are health-related behaviours – are quite different behaviours [38], the instrument comprises climate perceptions referring to these behaviours. The instrument assesses climate perceptions regarding daily eating and activity behaviour on the level of the individual. The individual rated statements concerning the whole family and was treated both as an observer and as a part of daily family life. Individuals were asked to state how health and health behaviours were valued within the family and how important these aspects are in daily life. Respondents were not asked to report their own behaviour or to communicate any personal feelings, and none of the questions were formulated in the first person. This approach is similar to, for instance, the measure of ‘Family Quality of Life’ [39] or the ‘Familienklimaskalen für Jugendliche’ [40].

The items were selected considering both the construct definition and previously developed measures of related constructs such as ‘family climate’ or ‘family environment’ [34,41-44] or ‘organizational health climate’ [37,45]. The items are assumed to reflect typical aspects of the social setting ‘family’ (e.g. communication, time together, encouragement of individuals, connectedness) and represent affective, cognitive, and instrumental facets of the family environment [31]. Considering the specificity of different health behaviours, the instrument comprises two separate scales: the Family Health Climate for Physical Activity (FHC-PA) and the Family Health Climate for Nutrition (FHC-NU). A first preliminary version consisted of 46 items for physical activity and nutrition.

An expert rating (n = 5) and a novice rating (n = 26) were conducted, and redundant items as well as items that did not fit the construct definition were removed. In the FHC-PA and FHC-NU Scale 26 and 22 items, respectively, were eliminated. Several items were rephrased.

Subsequently, the two scales with the remaining 20 (FHC-PA) and 24 (FHC-NU) items were psychometrically tested in two independent studies. In Study A N = 479 (70.8% female; age: M = 40.70, SD = 11.14, range 19 to 64 years) employees of the University of Konstanz (recruited via mail) completed the questionnaire. In Study B N = 167 families were recruited in 7 schools. All families comprised at least one child and one parent in the same household. Questionnaires from n = 167 children and n = 217 parents (51.6% female; age: M = 45.28, SD = 5.17, range 33 to 61) were available. For the subsequent explorative analyses only the data of the parents were used. The factorial structure was examined with exploratory factor analyses (principal axis factoring with oblique Promax rotation) indicating multidimensionality of both scales. Non-fitting items (FHC-PA 3 items, FHC-NU 4 items) were removed based on the following criteria: factor loading < .40, cross-loading > .30, communality < .30 and corrected item-scale correlation < .30 [46]. In the next step the instrument was extensively modified. Considering construct definition and multidimensionality of the scales, 13 (FHC-PA) and 10 items (FHC-NU) were added to create a sufficiently large item pool for explorative analyses and scale refinement in the subsequent step. The resulting version comprised 30 items for FHC-PA and FHC-NU, respectively. (Supplementary material is available from the authors).

## Study 1: refinement and validation of the factorial structure

Study 1 was conducted to explore the factorial structure, to refine the scale, and to cross-validate its factorial structure.

## Methods

The study conformed to the Declaration of Helsinki and the ethics guidelines of the German Psychological Society.

### Participants

Participants were volunteers from the community who were asked to fill in either a paper-and-pencil or an online version of the questionnaire. Whereas from the former written informed consent was obtained, the latter agreed to participate in the study by answering the questions after being presented the same informed consent in the preamble of the survey. Overall, 787 participants completed the questionnaire with the FHC-Physical Activity Scale and the FHC-Nutrition Scale. The participants’ mean age was 48.5 years (SD = 11; range 18 to 69 years), and 51% were female. 691 (87.8%) of the participants lived in a partnership at the time of the study and 725 (92.1%) had at least one child living in the household. Two-hundred and ninety-six (37.6%) participants had a university-entrance diploma (‘Abitur’).

### Measures

#### Demographics

Age, gender, and number of children were assessed by single questions. Marital status was dichotomized into ‘living alone’ and ‘living in a partnership/marriage’. Education level was assessed by asking for the highest school qualification. According to the German school system the categories ranged from ‘no qualification’ to ‘university-entrance diploma’ (‘Abitur’).

#### Family health climate

The FHC-PA and the FHC-NU were assessed with the first version of the FHC-Scales. The items were introduced with the item stem ‘In our family…’ , and answers were given on a four-point rating scale (0 = ‘definitely false’ , 1 = ‘rather false’ , 2 = ‘rather true’ , 3 = ‘definitely true’). The FHC-PA and the FHC-NU Scale both consisted of 30 items. The FHC-PA Scale contained items like ‘…we enjoy our time as a family doing physical activity (e.g. bike tours, hikes)’ or ‘…we make a point of being physically active during everyday life’. The FHC-NU Scale consisted of items like ‘…everybody enjoys having meals together’. or ‘…we talk about how to eat healthfully’.

### Data analysis

The data set was randomly divided into two samples. The first random sample (random sample I) was used to conduct exploratory factor analyses with SPSS 21® using principal axis factoring with oblique Promax rotation [47,48]. Separate analyses were carried out for the physical activity scale and the nutrition scale.

Confirmatory factor analyses were performed with AMOS 21® using the maximum-likelihood method with second random sample (random sample II). The commonly recommended fit indices *χ*^2^/df, CFI, SRMR and RMSEA were used to assess the goodness of fit. A good fit is indicated by 0 ≤ *χ*^2^/df ≤ 2, .97 ≤ CFI ≤ 1, 0 ≤ SRMR ≤ .05 and RMSEA ≤ .05, while values 2 < *χ*^2^/df ≤ 3, .95 ≤ CF < .97, .05 < SRMR ≤ .10 and .05 < RMSEA ≤ .08 indicate an acceptable fit [49].

The measurement invariance was tested using multi-group analyses. Both the chi-square difference statistic for testing invariance and the recommendations of Chen [50] were used. For loading invariance, Chen recommended for sample sizes of N > 300 with equal sample sizes that a change in CFI ≥ -.010 with a change in RMSEA ≥ .015 or a change in SRMR ≥ .030 indicates non-invariance [50].

Less than 5% of values for all variables were missing. Missing data were imputed using the Expectation Maximization algorithm in SPSS 21® after checking that missing values were completely at random using Little’s MCAR test [51]. Item distributions were inspected for multivariate normality. Skewness and excess of all items were below the thresholds of 2 and 7, respectively, as suggested by Curran, West, and Finch [52]. Multi-group analyses were conducted to test measurement invariance.

## Results

### Exploratory factor analysis and refinement of the FHC-scales

Exploratory factor analyses were used to explore the latent structure. For both item sets, the requirements for exploratory factor analysis in this sample were fulfilled (FHC-PA: Kaiser-Meyer-Olkin = .96, Bartlett’s test of sphericity *χ*^2^(325) = 7222.00, p < .01; FHC-NU: Kaiser-Meyer-Olkin = .95, Bartlett’s test of sphericity *χ*^2^(325) = 7027.59, p < .01). There were no correlations above .85 between any pair of items. For the FHC-PA Scale, the Kaiser-criterion (eigenvalue > 1) yielded four factors with eigenvalues greater than one. Parallel Analysis of the eigenvalues suggested three factors, and a MAP Test suggested four factors. For the FHC-NU Scale, Kaiser-criterion suggested three factors, parallel analysis and MAP Test yielded four factors. Using the initial factor solutions, items were removed step by step based on the following criteria: factor loading < .40, cross-loading > .30, communality < .30 and corrected item-scale correlation < .30 [46]. According to these criteria 16 items were removed from the FHC-PA Scale and 13 items were removed from the FHC-NU Scale. Finally, three factors for the physical activity scale and four factors for the nutrition scale were extracted.

#### FHC-physical activity scale

The FHC-PA Scale consists of three factors: *value* (5 items, eigenvalue = 7.33), *cohesion* (5 items, eigenvalue = 1.55), and *information* (4 items, eigenvalue = 1.02). The three factors accounted for 70.68% of the variance. The factor *value* consists of items reflecting the importance of being physically active for the whole family. A high score implies that physical activity is part of family members’ daily life. *Cohesion* covers joint physical activities and having fun together during these activities. The search, sharing, and use of information related to sports and exercise is captured by the factor *information*. Table 1 shows the internal consistencies and the associated items with means, standard deviations, item-scale correlations and factor loadings for both random samples for the three factors (see also Additional file 2: German version of FHC-Scales). The factor loadings range from .58 to .90. All three factors showed good internal consistencies ranging from .81 to .91.

**Table 1:** FHC-PA – factors and item parameters for study 1 and study 2

**Factor**		Item*	**Sample**								
			**Study 1 – sample I (n = 389)**			**Study 1 – sample II (n = 398)**			**Study 2 (n = 210)**		
		**In our family…**	**M (SD)**	**r**_**it**_	**a**	**M (SD)**	**r**_**it**_	**a**^**1**^	**M (SD)**	**r**_**it**_	**a**^**1**^
Value	[1]	…we make a point of being physically active during daily life.	1.72 (.79)	.75	.69	1.78 (.78)	.77	.81	1.96 (.73)	.69	.73
α_1-I_ = .91	[2]	…it is normal to be physically active on a regular basis.	1.84 (.82)	.74	.77	1.92 (.84)	.76	.80	2.07 (.80)	.80	.85
α_1-II_ = .92	[3]	…it goes without saying that we exercise and are physically active on a regular basis.	1.75 (.87)	.81	.90	1.86 (.89)	.85	.89	2.09 (.88)	.82	.86
α_2_ = .90	[4]	…it is normal to be physically active in our leisure time.	1.80 (.76)	.79	.79	1.92 (.81)	.84	.89	2.02 (.86)	.75	.80
	[5]	…we agree that physical activities are part of daily life.	1.90 (.81)	.73	.70	1.97 (.80)	.75	.80	2.02 (.79)	.73	.78
Cohesion	[1]	…we like being together during physical activities (e.g. bike tours, hikes).	1.81 (.91)	.66	.75	1.76 (.89)	.63	.60	1.97 (.85)	.71	.67
α_1-I_ = .90	[2]	…we enjoy exercising together.	1.45 (.88)	.75	.68	1.51 (.90)	.73	.78	1.56 (.85)	.81	.88
α_1-II_ = .90	[3]	…we have fun doing physical activities together (e.g. bike tours, hikes).	1.81 (.89)	.78	.83	1.89 (.90)	.76	.77	2.00 (.89)	.77	.74
α_2_ = .91	[4]	…we find it very pleasant to be physically active together.	1.64 (.84)	.80	.75	1.68 (.88)	.80	.89	1.76 (.84)	.72	.80
	[5]	…we like spending time together in sports activities.	1.56 (87)	.82	.88	1.59 (.87)	.82	.90	1.69 (.86)	.84	.90
Information	[1]	…we watch TV-programmes on physical activity and exercise.	1.30 (.84)	.52	.58	1.40 (.89)	.67	.72	1.15 (.96)	.58	.65
α_1-I_ = .81	[2]	…we explicitly look for the latest information on physical activity and exercise to stay up to date.	1.12 (.80)	.59	.58	1.16 (.84)	.69	.80	.97 (.80)	.64	.74
α_1-II_ = .86	[3]	…we collect information (e.g. on the internet) on physical activity and exercise.	1.07 (.74)	.72	.88	1.18 (.78)	.74	.81	.93 (.83)	.71	.81
α_2_ = .83	[4]	…we read newspaper or magazine articles on fitness, physical activity, and exercise.	1.40 (.85)	.68	.80	1.42 (.86)	.74	.80	1.33 (.88)	.69	.77

*M* – means, *SD* – standard deviations, r_it_ – corrected item-scale correlations, a – factor loadings of EFA, a^1^– factor loadings of CFA.

*The German version of the FHC-Scale is shown in Additional file 2.

#### FHC-nutrition scale

The FHC-NU Scale consists of four factors: *value* (4 items, eigenvalue = 6.84), *cohesion* (5 items, eigenvalue = 2.70), *communication* (5 items, eigenvalue = 1.39) and *consensus* (3 items, eigenvalue = .92). The factors accounted for 69.67% of the variance. The FHC-NU Scale showed a different factorial structure. Similarly to the FHC-PA, the factor *value* captures the family’s emphasis on a health enhancing nutrition in daily life. *Cohesion* is reflected in common family meals and the importance of eating together with other family members. In contrast to the FHC-PA Scale, there is no information factor but the factors *communication* and *consensus*. The factor *communication* encompasses that nutrition is a natural content of conversations and that family members support each other concerning a balanced diet. *Consensus* reflects that family members agree with each other in aspects related to daily eating behaviour. The internal consistency of the factors was good, ranging from .74 (consensus, 3 items) to .90. The factor loadings ranged from .62 to .82 (see Table 2).

**Table 2:** FHC-NU – factors and item parameters for study 1 and study 2

**Factor**		Item*	**Sample**								
			**Study 1 – sample I (n = 389)**			**Study 1 – sample II (n = 398)**			**Study 2 (n = 210)**		
		**In our family…**	**M (SD)**	**r**_**it**_	**a**	**M (SD)**	**r**_**it**_	**a**^**1**^	**M (SD)**	**r**_**it**_	**a**^**1**^
Value	[1]	…a healthy diet plays an important role in our lives.	1.92 (.78)	.71	.74	2.01 (.73)	.69	.76	2.22 (.74)	.64	.75
α_1-I_ = .90	[2]	…we naturally pay attention to eating healthfully.	1.86 (.76)	.80	.78	1.93 (.73)	.74	.81	2.10 (.71)	.70	.79
α_1-II_ = .88	[3]	…we routinely eat healthfully.	1.93 (.78)	.76	.71	1.99 (.70)	.76	.83	2.22 (.65)	.67	.75
α_2_ = .84	[4]	…it is normal to choose healthful foods.	1.95 (.78)	.80	.80	1.98 (.70)	.73	.80	2.30 (.67)	.70	.76
Communication	[1]	…we are interested in articles (e.g. in magazines) on healthful nutrition.	1.43 (.85)	.63	.71	1.51 (.84)	.62	.66	1.55 (.92)	.56	.63
α_1-I_ = .86	[2]	…we remind each other to pay attention to a healthful diet.	1.65 (.81)	.66	.69	1.76 (.78)	.71	.78	1.73 (.83)	.66	.75
α_1-II_ = .86	[3]	…we talk about which foods are healthful.	1.84 (.84)	.67	.63	1.95 (.79)	.66	.75	2.35 (.78)	.55	.63
α_2_ = .82	[4]	…we support each other to refrain from unhealthful things.	1.61 (.74)	.61	.58	1.63 (.75)	.65	.71	1.82 (.82)	.55	.64
	[5]	…we talk about how to eat healthfully.	1.69 (.85)	.78	.93	1.73 (.81)	.75	.81	1.89 (.83)	.72	.81
Cohesion	[1]	…we appreciate spending time together during meals.	2.46 (.66)	.71	.75	2.47 (.66)	.73	.79	2.65 (.55)	.75	.85
α_1-I_ = .89	[2]	…everybody enjoys having meals together.	2.42 (.71)	.71	.74	2.44 (.64)	.72	.78	2.63 (.57)	.64	.66
α_1-II_ = .89	[3]	…eating together is a part of our daily family life.	2.30 (.76)	.78	.82	2.36 (.73)	.77	.83	2.64 (.62)	.61	.65
α_2_ = .87	[4]	…we enjoy meals most when we sit at the same table.	2.36 (.73)	.74	.80	2.39 (.72)	.72	.76	2.63 (.58)	.72	.81
	[5]	…we try to eat together as often as possible.	2.38 (.75)	.70	.77	2.42 (.73)	.73	.76	2.68 (.56)	.75	.82
Consensus	[1]	…we rarely argue about food- or diet-related matters.	1.99 (.86)	.48	.62	2.03 (.90)	.41	.44	1.90 (.97)	.33	.39
α_1-I_ = .74	[2]	…we agree on diet and nutrition.	1.90 (.79)	.62	.62	1.88 (.76)	.63	.88	1.95 (.77)	.61	.89
α_1-II_ = .73	[3]	…we usually agree on meals and food choices.	1.88 (.77)	.61	.65	1.93 (.75)	.65	.81	1.97 (.84)	.54	.74
α_2_ = .67											

*M* – means, *SD* – standard deviations, r_it_ – corrected item-scale correlations, a – factor loadings of EFA, a^1^– factor loadings of CFA.

*The German version of the FHC-Scale is shown in Additional file 2.

### Cross-validation of the FHC-scales

The factorial structure was cross-validated in random sample II using confirmatory factor analyses (CFA). Subsequently, the invariance of the model was tested using multi-group analyses with random samples I and II, women and men, and older (≥50 years) and younger (< 50 years) participants, respectively. Invariance across different groups is an indicator for measurement quality and validity. If the measurement model is equivalent the instrument measures the construct in the same way across different samples. The validity of a measure requires metrical invariance of the measurement model and equal factor loadings across different groups [53,54].

#### FHC physical activity

**Figure 1:**
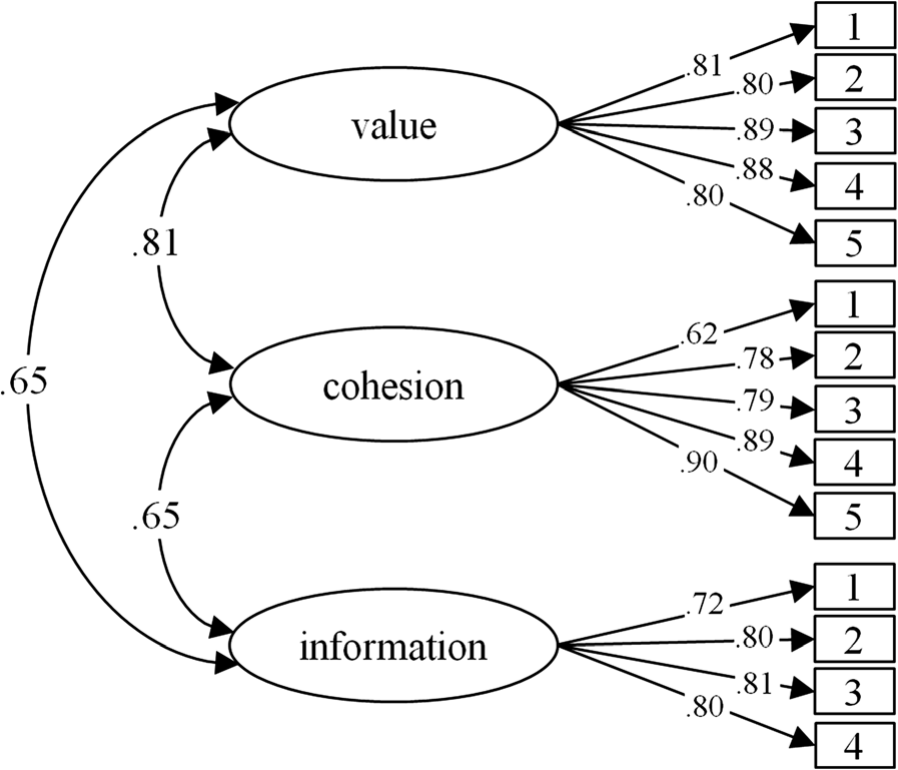
FHC-PA – standardized factor loadings and interfactor correlations.

Means, standard deviations, and item-scale correlations for random sample II are shown in Table 1. The fit indices *χ*^2^ = 222.69, df = 74, p < .01; *χ*^2^/df = 3.01; CFI = .96; SRMR = .04; RMSEA = .07, CI .06/.08 indicated an acceptable global fit for the assumed model. The model with factor loadings and factor correlations are shown in Figure 1. In random sample II, the factors were moderately intercorrelated (r_cohesion-information_ = .65, r_value-information_ = .65, p < .01) except for *cohesion* and *value* with r = .81.

Invariance tests with the model shown in Figure 1 indicate that the model is equivalent across different subsamples. Comparing samples I and II, the constraint of equal factor loadings did not reduce the measurement properties, and the two models did not differ significantly (Δχ^2^ = 11.92, Δdf = 11, p = .37). The fit indices for the unconstraint and constraint models are shown in Table 3. The model with equal factor loadings across females and males did not differ from the unconstrained model (Δχ^2^ = 12.69, Δdf = 11, p = .31). Across age groups both the unconstrained model and the constrained model had an acceptable fit and did not differ in goodness of fit (Δχ^2^ = 13.27, Δdf = 11, p = .28).

**Table 3:** Fit-Indices of the unconstraint and constraint models of the FHC-scale

**FHC-PA**										
**Samples**	**Model**	***χ***^**2**^	**df**	**p**	***χ***^**2**^**/df**	**CFI**	**SRMR**	**RMSEA**	**90% CI**	**AIC**
I (n = 389) & II (n = 398)	Unconstrained	327.809	148	<.001	2.215	.976	.0316	.039	.034/.045	507.809
	Constrained	339.636	159	<.001	2.136	.976	.0316	.038	.032/.045	497.636
men (n = 385) & women (n = 401)	Unconstrained	352.809	148	<.001	2.384	.972	.0389	.042	.036/.048	532.809
	Constrained	363.169	159	<.001	2.284	.972	.0400	.040	.035/.046	521.169
< 50 (n = 424) & ≥ 50 (n = 363)	Unconstrained	360.352	148	<.001	2.435	.971	.0367	.043	.037/.048	540.352
	Constrained	373.557	159	<.001	2.349	.971	.0386	.041	.036/.047	531.557
**FHC-NU**										
I (n = 389) & II (n = 398)	Unconstrained	490.796	226	<.001	2.172	.963	.0409	.039	.034/.043	718.796
	Constrained	502.027	239	<.001	2.101	.964	.0413	.037	.033/.042	704.027
men (n = 385) & women (n = 401)	Unconstrained	508.458	226	<.001	2.250	.96	.0443	.040	.035/.045	736.458
	Constrained	512.834	239	<.001	2.146	.961	.0446	.038	.034/.043	714.834
< 50 (n = 424) & ≥ 50 (n = 363)	Unconstrained	517.825	226	<.001	2.291	.960	.0431	.041	.036/.045	745.825
	Constrained	533.392	239	<.001	2.232	.959	.437	.040	.035/.044	735.392

#### FHC nutrition

**Figure 2:**
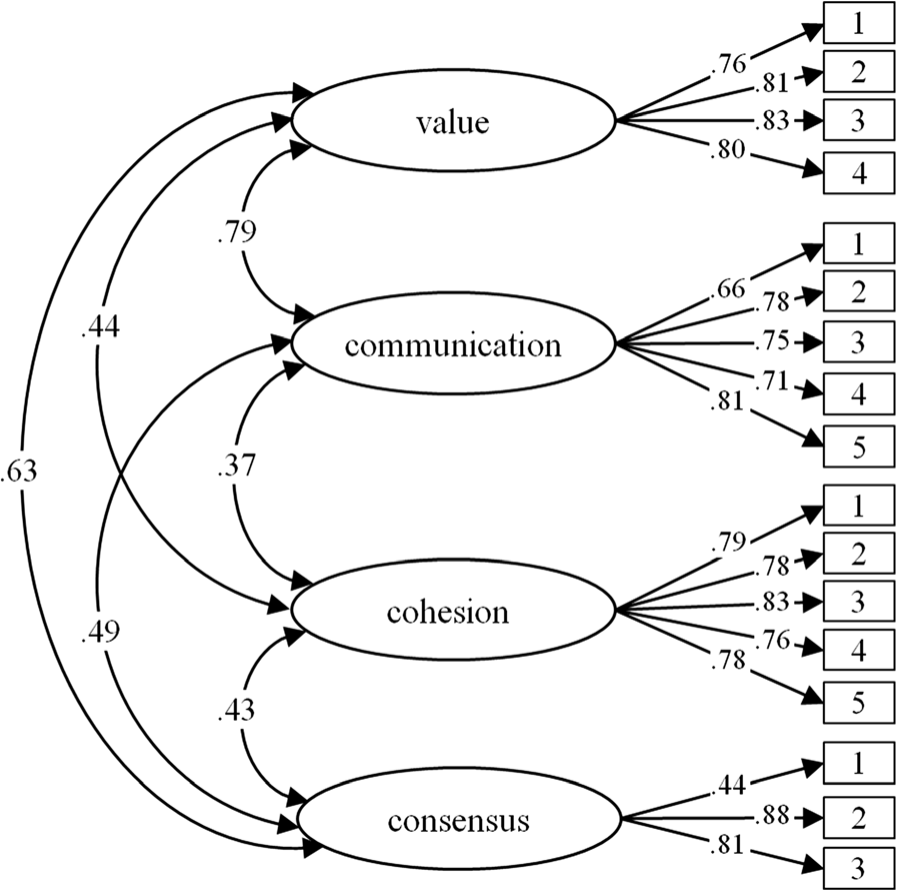
FHC-NU – standardized factor loadings and interfactor correlations.

The means, standard deviations, and item-scale correlations for the random sample II are shown in Table 2. The latent factor structure of the FHC-NU is shown in Figure 2. The indices for the model indicate an acceptable fit (*χ*^2^ = 278.30, df = 113, p < .01, *χ*^2^/df = 2.46, CFI = .96; SRMR = .04; RMSEA = .06, CI .05/.07). The factors were moderately intercorrelated (r = .37-.63, p < .01), except for the correlation between *value* and *communication* with r = .79.

The model revealed invariance between the random samples. The fit for an unconstrained model with simultaneous modelling of random samples I and II is acceptable (see Table 3). The constraint of equal factor loadings did not reduce the model fit (Δχ^2^ = 11.23, Δdf = 13, p = .59). Measurement invariance was confirmed for females and males. The constrained model with equivalent factor loadings fitted the data as well as the unconstrained model (Δχ^2^ = 4.38, Δdf = 13, p = .99). An invariance test across age groups indicated that the factor loadings were equal for both the younger and the older participants. The constrained model fitted the data as well as the unconstrained model (Δχ^2^ = 15.57, Δdf = 13, p = .27).

## Study 2: construct validation of the final version

A second study was conducted to investigate the construct validity by testing the assumed correlations between FHC and cognitive, motivational, and behavioural variables from the individual perspective as well as from the perspective of interrelated family members.

## Methods

### Procedure

Participants were recruited in twelve schools in the district of Konstanz, Germany. After making an appointment with the schools’ principals the classes were visited. The students were informed about the aims and requirements of the ‘Family and Health-Study’ and received an envelope with three questionnaires, one for themselves, one for their mothers, and one for their fathers. The students were asked to forward the questionnaires to their parents. Within one week, the children and their parents completed the questionnaires and returned them to their teachers. The teachers collected the envelopes and gave them to the principals where the envelopes were picked up. The study conformed to the Declaration of Helsinki and the ethics guidelines of the German Psychological Society and written informed consents were obtained from the parents of the participating students.

### Participants

Three-hundred and nineteen families filled out the questionnaires. Since the individual perspective as well as the perspective of interrelated family members was taken into account, a subsample of this cohort was used for questionnaire validation. Families where child, mother, and father completed the questionnaires and currently live in the same household were included. The subsample consisted of 210 parents (210 mothers and 210 fathers). The women had a mean age of 45.1 years (SD = 4.3; range 34 to 45 years). Sixty-five (31%) women had a university-entrance diploma (‘Abitur’) and 23 (11%) had an advanced technical college certificate (‘Fachhochschulreife’). At the time of the study, 33 (15.7%) worked full-time, 141 (67.1%) worked part-time, 2 (1%) were unemployed or retired, 2 (1%) were on parental leave, 23 (11%) were homemakers and 8 (3.8%) were freelancers. On average parents had lived for 18.5 years (SD = 6.08, range 1 to 35 years) in a joint household with their spouses. All parents had at least one child aged 12 to 24 years (M = 14.2, SD = 1.6 years) that lived in the same household. The men had a mean age of 47.6 (SD = 6.7, range 21 to 74 years). Seventy-eight (37.1%) men had a university-entrance diploma (‘Abitur’) and 41 (19.5%) had an advanced technical college certificate (‘Fachhochschulreife’). At the time of the study, 187 (89%) men worked full-time, 7 (3.3%) worked part-time, 1 was on parental leave, 6 (2.9%) were unemployed or retired, 4 (1.9%) were homemakers and 5 (2.4%) were freelancers.

### Measures

#### Demographics

Age, gender, and age of the children living in the household were assessed by single questions. Marital status was categorized into ‘living alone’ and ‘living in a partnership/marriage in the same household’ and ‘living in a partnership/marriage not in the same household’. Education level was assessed by asking for the highest school qualification. According to the German school system the categories ranged from ‘no qualification’ to ‘university-entrance diploma’ (‘Abitur’). Employment status was categorized in ‘full-time’, ‘part-time’, ‘in parental leave’, ‘homemakers’, ‘unemployed’, ‘retired’, and ‘freelancer’.

#### Family health climate

The final versions of both Family Health Climate Scales were used. The FHC-PA Scale consists of three subscales (value, cohesion, and information) and a total of 14 items. The FHC-NU Scale is comprised of four subscales (value, cohesion, communication, and consensus) and a total of 17 items. The internal consistencies are listed in Tables 1 and 2.

#### Self-determination

Three scales of the German version of the Behavioural Regulation of Exercise Questionnaire 2 (BREQ-2) were used to measure self-determination of exercise: identified (3 items, e.g. ‘I value the benefits of exercise’) and intrinsic regulation (4 items, e.g. ‘I exercise because it’s fun’), and amotivation (4 items, e.g. ‘I don’t see why I should have to exercise’) [55]. The responses were scored on a 4-point scale ranging from 0 = ‘not true’ to 3 = ‘true’. The internal consistencies were acceptable to good for these subscales (intrinsic: α = .91; identified: α = .75; amotivation: α = .80).

Self-determination of healthy eating was measured with three scales of the German version of the Regulation of Eating Behaviour Scale (REBS) [56]. Responses were scored on the same a 4-point scale as described above. The internal consistency for the scale identified motivation (4 items, e.g. ‘I believe it will eventually allow me to feel better’) was acceptable (α = .75). Coefficient alphas for the scales intrinsic motivation (4 items, e.g. ‘I take pleasure in fixing healthy meals’) and amotivation (4 items, e.g. ‘I can’t really see what I’m getting out of it’) were low (α = .63 and α = .67, respectively).

#### Routines – family meals and joint physical activities

Family meals are an important behavioural routine in daily family life. The frequency of family meals was assessed by the question ‘on how many days per week does the family have at least one joint meal per day’. Participants rated the frequency on a 5-point scale by indicating never, 1–2 times a week, 3–4 times a week, 5–6 times per week or every day. The behavioural routine ‘to do joint physical activities’ was assessed by asking how often the spouses engage in physical activities together and how often they engage in physical activities with the child. The answers were given on a 5-point scale ranging from 0 = ‘never’ to 4 = ‘very frequently’.

#### Interactions – social support between family members

Support of physical activity and healthy eating between family members was assessed with three questions each. The participants rated how often they support their child to engage in physical activities (to eat healthy), how often they support their spouse, and how often they receive support from other family members. Answers were given on a 5-point scale ranging from 0 = ‘not at all’ to 4 = ‘very frequently’.

#### Availability of healthy and unhealthy foods

Respondents were asked to rate how often healthy (fruits and vegetables) and unhealthy food items (soft drinks, fast food) are available in the household using a 5-point rating scale with the categories 0 = ‘never’, 1 = ‘rarely’, 2 = ‘sometimes’, 3 = ‘frequently’ and 4 = ‘always’.

### Data analysis

Less than 5% of values were missing for all variables. Missing data were imputed using the Expectation Maximization algorithm in SPSS 21® after checking that missing values were completely at random using Little’s MCAR test [51]. Item distributions were inspected for multivariate normality. Skewness and excess of all items were below the thresholds of 2 and 7, respectively, as suggested by Curran, West, and Finch [52].

## Results

### FHC physical activity

The FHC-PA Scale was cross-validated with this sample [46,57]. The model fit was acceptable (*χ*^2^ = 285.80, df = 74, p < .01; *χ*^2^/df = 3.86; CFI = .94; SRMR = .04; RMSEA = .08, CI .07/.09).

Means, standard deviations, corrected item-scale correlations, and factor loading are listed in Table 1.

All three subscales of the FHC-PA and the aggregated scale (FHC-PA agg) showed the hypothesized correlations with self-determined physical activity, joint activities within the family and social support (see Table 4).

**Table 4:** Correlations between FHC-PA and self-determination, joint activities, and support of physical activity

		**Value**	**Cohesion**	**Information**	**Aggregated**
Self-determination	Intrinsic	.62 (<.001)	.45 (<.001)	.24 (<.001)	.56 (<.001)
	Identified	.60 (<.001)	.36 (<.001)	.27 (<.001)	.52 (<.001)
	Amotivation	-.35 (<.001)	-.23 (<.001)	-.08 (.114)	-.28 (<.001)
Joint activities	With child	.40 (<.001)	.57 (<.001)	.22 (<.001)	.51 (<.001)
	With partner	.46 (<.001)	.45 (<.001)	.19 (<.001)	.48 (<.001)
Social support	Child	.30 (<.001)	.27 (<.001)	.21 (<.001)	.32 (<.001)
	Partner	.32 (<.001)	.29 (<.001)	.16 (.001)	.35 (<.001)
	Received	.32 (<.001)	.31 (<.001)	.14 (.005)	.35 (<.001)

#### Self-determination – individual

The individual’s perception of the FHC-PA is significantly correlated with individual self-determination (p < .01). The more positively the FHC-PA is perceived, the stronger intrinsic (r _FHC-PA agg_ = .56) and identified (r _FHC-PA agg_ = .52) motives regulate the activity behaviour and the less the persons are amotivated to exercise (r _FHC-PA agg_ = -.28).

#### Self-determination – interrelated family members

Further analyses showed interindividual correlations within the parent dyads (p < .01). The more positively the mother values the FHC-PA, the higher the father rated his intrinsic (r _FHC-PA agg_ = .38) and identified motivation (r _FHC-PA agg_ = .40) to exercise. The same pattern appeared for the perception of the FHC of the father and the self-determination of the mother. The more positively the father valued the FHC-PA the higher the mother rated her intrinsic (r _FHC-PA agg_ = .48) and identified motivation (r _FHC-PA agg_ = .43).

#### Joint activities and social support – individual

The perception of the FHC-PA significantly correlated with the frequency of joint activities and social support between the family members (p < .01). A positive perception of FHC-PA was associated with a higher frequency of joint activities with the child (r _FHC-PA agg_ = .51) and the partner (r _FHC-PA agg_ = .48), more support of the child (r _FHC-PA agg_ = .32) and the partner (r _FHC-PA agg_ = .35) and more received support from other family members (r _FHC-PA agg_ = .35, see Table 4).

#### Joint activities – interrelated family members

The individual’s perception of the FHC-PA also correlated with the corresponding rating of the spouse (p < .01). A positive perception of the mother is associated with more frequent joint activities with the spouse rated by the father (r _FHC-PA agg_ = .26) and vice versa (r _FHC-PA agg_ = .28).

### FHC nutrition

The FHC-NU Scale was cross validated with the sample of Study 2. The fit was acceptable (*χ*^2^ = 249.553, df = 113, p < .01; *χ*^2^/df = 2.208; CFI = .956; SRMR = .050; RMSEA = .054, CI .045/.063).

Means, standard deviations, corrected item-scale correlations, and factor loadings are listed in Table 2.

The hypothesized relationships were found for the subscales and the aggregated scale (FHC-NU agg).

#### Self-determination – individual

The perception of FHC-NU was positively related to intrinsic and identified motivation and negatively correlated to amotivation (p < .01, see Table 5). The more positively the FHC-NU was perceived, the higher was the self-determination of healthy eating (FHC-NU agg: r_intrinsic_ = .42, r_identified_ = .47, r_amotivation_ = -.38).

**Table 5:** Correlations between FHC-NU and self-determination, food environment and support of healthy eating

		**Value**	**Cohesion**	**Communication**	**Consensus**	**Aggregated**
Self-determination	Intrinsic	.32 (<.001)	.18 (<.001)	.48 (<.001)	.15 (.002)	.42 (<.001)
	Identified	.41 (<.001)	.18 (<.001)	.47 (<.001)	.20 (<.001)	.47 (<.001)
	Amotivation	-.37 (<.001)	-.28 (<.001)	-.26 (<.001)	-.11 (.027)	-.38 (<.001)
Food environment	Joint meals^1^	.20 (<.001)	.33 (<.001)	.10 (.042)	.06 (.236)	.24 (<.001)
	Availability vegetables^1^	.26 (<.001)	.16 (.001)	.22 (<.001)	.14 (.006)	.27 (<.001)
	Availability soft drinks^1^	-.31 (<.001)	-.10 (.043)	-.31 (<.001)	-.11 (.025)	-.31 (<.001)
Social support	Child	.18 (.001)	.14 (.004)	.33 (<.001)	-.09 (.061)	.24 (<.001)
	Partner	.16 (.002)	.12 (.019)	.33 (<.001)	-.04 (.455)	.22 (<.001)
	Received	.09 (.094)	.07 (.186)	.30 (<.001)	-.02 (.719)	.16 (.002)

^1^Spearman’s correlation coefficient.

#### Self-determination – interrelated family members

This correlation pattern was also found between the mother’s perception of the FHC-NU and the father’s self-determination and vice versa. If the FHC-NU was valued positively, the spouse rated his/her intrinsic or identified motivation as high and his/her amotivation as low (mother’s FHC-NU agg and father’s self-determination: r_intrinsic_ = .25, r_identified_ = .32, p < .01, r_amotivation_ = -.08, p = .20; father’s FHC-NU agg and mother’s self-determination: r_intrinsic_ = .33, r_identified_ = .27, p < .01, r_amotivation_ = -.17, p < .05).

#### Joint meals and availability of vegetables and soft drinks – individual

The perception of the FHC-NU correlated with the frequency of family meals and the availability of vegetables and soft drinks (p < .01). A positive perception was related to more frequent joint meals (r _FHC-NU agg_ = .42), a higher availability of vegetables (r _FHC-NU agg_ = .47) and a lower availability of soft drinks (r _FHC-NU agg_ = -.38, see Table 5).

#### Joint meals and availability of vegetables and soft drinks – interrelated family members

These correlations were also found interindividually (p < .01). The more positively the mother valued the FHC-NU, the higher the father rated the frequency of family meals (r _FHC-NU agg_ = .20) and the availability of vegetable (r _FHC-NU agg_ = .32) and the lower he rated the availability of soft drinks (r _FHC-NU agg_ = -.26).

#### Social support – individual

Finally, the perception of the FHC-NU was associated with the amount of social support the person gave and received (p < .01). The more positively the person valued the FHC, the more often she supported the spouse (r _FHC-NU agg_ = .22) and the child (r _FHC-NU agg_ = .24) and the more support she received from them (r _FHC-NU agg_ = .16, see Table 5).

## Discussion

Individual behaviour is embedded in a social context, and relationship factors influence health behaviour [58]. Thus, for explaining an individual’s health behaviour the social environment needs to be considered. The family is the most stable and hence probably the most important social environment [2,59]. This study introduces a new approach to describing and measuring the influence of family on individual eating and physical activity behaviour. The aim of this study was to develop a psychosocial construct that fits the family-as-system approach. We suggest that the Family Health Climate is a family level variable that affects the health behaviour of family members. Two scales were developed measuring the climate concerning healthy eating (FHC-NU Scale) and physical activity (FHC-PA Scale). The respondents are addressed as observers and as parts of their family and asked to state their perception of the family as a whole. Both scales are multifaceted: the FHC-PA Scale reflects the factors *value*, *cohesion*, and *information*, and the FHC-NU Scale consists of the four factors *value*, *cohesion*, *communication*, and *consensus*. The psychometric quality of both scales was good, both scales showed an acceptable fit and measurement invariance across different samples. The relationships to relevant variables indicated good construct validity at the intraindividual and interindividual level.

### FHC-PA

FHC-PA showed the assumed relations to an individual’s self-determination and to relationships and interactions within the family such as provided and received support, and joint physical activity. The strengths of the relationships varied with the weakest correlations for *information* and the strongest for *value*, which may be due to the fact that the latter is the most general factor while the other factors are more specific. With regard to joint activities, the factors *cohesion* and *value* showed comparable correlation patterns.

Importantly, these correlations were found at the individual and interindividual level. For example, the perceived FHC of the mother correlates significantly with the self-determination of the father, and the more positive the father valued the FHC the higher is the intrinsic and identified motivation of the mother. This result suggests the notion that the FHC-Scales actually measure the family level: the FHC is rated by the individual and this individual perception is associated with motivational and behavioural aspects of another person in the family. Therefore, the FHC reflects an aspect of the shared family environment and it could be assumed that the FHC affects the individual cognition, motivation, and behaviour as well as the cognition, motivation, and behaviour of other family members.

The three subscales, *values, cohesion,* and *communication,* were moderately to highly intercorrelated. We suggest that although the subscales *value* and *cohesion* showed a high intercorrelation, that they represent two aspects of the activity related FHC. While *value* reflects the perception of the shared valuation of the importance of exercising, *cohesion* refers to family routines and interactions. Depending on the research question and a wide or focused view on physical activity as dependent variable, the use of the three subscales (disaggregated model) or of the aggregated index may be appropriate [60,61]. The relationships to different facets of activity behaviour should be tested in further studies, and the contribution of the different dimensions of the FHC-PA could be analysed in this context.

### FHC-NU

Although a similar factorial structure for both health-related behaviours was assumed, there are obvious differences between the two aspects. Nutrition and physical activity are both part of daily family life but their appearance and integration into family life is different. Nutrition is more present in family life, most families have one kitchen, one freezer, a shared stock of foods, and there are more or less frequent family meals. The communication about nutrition seems to be inherent because of this everyday presence and these shared occasions, and it is obvious that there could be agreement and disagreement on nutrition-related aspects within the family.

The perceived FHC-NU was associated with self-determination, joint meals, availability of vegetables, and soft drinks, and both provided and received support. These correlations were not only found on the individual level but also between the spouses.

### Characteristics of the FHC

This new construct and the developed measures have three important characteristics.

1) *FHC is a family level variable.* For instance, a specific approach was chosen to create a family level variable. Previously studied family influences mostly refer to influences of one person (mostly a parent) on another person (mostly a child or adolescent) [62]. Some measures integrated physical components of the family environment and parental and child behaviour [63,64]. However, the use of the term ‘family’ in these contexts does not reflect a family level variable. There are some concepts that refer to the family as a whole, for example family cohesion, family environment, family climate, and family quality of life [39,41,43]. These concepts were applied to study children’s and adolescent’s well-being, depression, deviating behaviour, substance use, or school adjustment [22,65-67]. In the field of health behaviours, only few studies concerning alcohol and cigarette consumption of adolescents have shown associations to family connectedness [22]. Lacking family cohesion was found to be associated with breakfast skipping in adolescent girls [68], which goes along with a higher risk of being overweight or obese [10], and with adults’ adherence to medical treatment [69]. These results indicate that family level variables are important for individual health and health behaviour. The aim of the study was to close this gap and to create a variable that refers to the family. The results of both FHC-Scales show individual and interindividual relations to specific determinants of health behaviour and indicate that the FHC-Scales measure a family level construct. In addition to mothers’ and fathers’ ratings of FHC and determinants of health behaviour the ratings of the children should be included in further analyses to strengthen these results. In the next step it has to be shown that the FHC is associated with individual health behaviour. Furthermore, it would be interesting to take into account different scores of the FHC to predict individual health behaviour, the individual score as well as an aggregated score across family members [70].

2) *FHC and health behaviour.* The concepts described above are unspecific, and hence relations to the specific context of individual health behaviour, such as nutrition or physical activity, are expected to be low [22,33]. Another characteristic of this new construct and the developed measures is the focus on health and health behaviour. Daily family life includes many health-related cues [59]. Daily family routines, such as regularity of family meals, choice and preparation of food, and conversations about health-related topics are associated with health and health behaviour of family members [71]. The family is an entity of control and organization and provides strong socio-emotional support [72]. Specific formal or informal rules regulating smoking, eating, or activity and inactivity patterns may develop within a family [59]. Family life implies shared values, common interpretation patterns, and behavioural habits [73]. Concepts of health and illness, the perception of well-being, values, and attitudes towards one’s body, and the perception of competences in different areas, for example physical competences, are socialized in the family [2,74]. The aim of this study was to create a family level variable that addresses these family specific attributes related to health and health behaviour. The associations between the perception of the FHC and the ratings of family environmental aspects like joint meals, joint activities and availability of healthy and unhealthy food as well as characteristics of social interactions within the family (social support) indicate that the FHC addresses health related attributes of the family.

3) *FHC affects all family members.* A family level variable should not only capture health behaviour of children and adolescents but also the health behaviours of adults. Evidence that the family environment affects the adults living in the family is scarce. For example, it is well known that marital status is associated with mortality risk. Possible mechanisms that mediate this relationship are social control and social support of health behaviour as dimensions of the social integration associated with a marriage [75]. Most research on family influences has focused on social support or social control from family members [67,76,77]. Some studies have examined other family environmental factors. For instance, the quality and quantity of family meals do not only influence children’s body mass index but also the body mass index of the adults [78]. The results of this study indicate that the FHC is a family level variable that is associated with adults’ health behaviour. Future research should aim to further investigate such family influences on adult’s health behaviours.

### Limitations

The participants in both studies were volunteers, which may have biased the results. Both samples were more highly educated than the average German population [79] possibly limiting the generalizability of the findings. Therefore, replicating the results in other samples or cultures is desirable. Moreover, the relationship between FHC and socioeconomic status should be investigated. Although the psychometric properties and the construct validity are satisfying, there are some deficiencies. For instance, the scales should be improved to eliminate the correlation between the factors in the FHC-PA Scale and to elaborate if there are some additional facets of the FHC climate.

The construct validity was demonstrated by the strong correlations between the perception of the FHC and the self-determination of healthy eating and physical activity. On the individual level measurement biases cannot be excluded as awareness or consciousness regarding healthful everyday behaviour could affect both self-determination and perception of the FHC. However, the same correlations were found across ratings from different individuals (interindividual level): The more positive one person (e.g. the mother) rated the FHC, the higher the intrinsic motivation was rated by another person (e.g. the father). These interindividual associations render systematic biases rather unlikely.

Further examination of the measure in different samples would provide greater confidence in the psychometric properties. The test-retest reliability was not tested in these studies; therefore it is not possible to make statements about stability over time.

## Conclusions

The family is an important social context that affects individual health behaviour. Extensive research has aimed to examine the influences of the family environment on eating and activity behaviour. In this study, a new approach was adopted aiming to develop a construct that refers to the family as a whole and represents an attribute of the whole family by reflecting a family environmental aspect related to health and health behaviour. The Family Health Climate represents a family level variable, which is assumed to affect the health behaviour of the family members. Two scales were developed that measure the climate with regard to healthy eating and physical activity. The scales have good psychometric quality, and encouraging results for construct validity were found. The inventory may be useful in studies examining the influence of the family environment on individual health behaviour (eating behaviour and physical activity) and in those investigating healthy and unhealthy family environments. Depending on the research focus, the scales can be used in combination or separately. Merging previous results on family influences with the new approach Family Health Climate and combing these with developmental and family psychological perspectives will surely result in advances in understanding the complexity of family influences on individual health behaviour.

## Abbreviations

FHC: Family health climate; FHC-PA: Family health climate for physical activity; FHC-NU: Family health climate for nutrition.

## Competing interests

The authors declare that they have no competing interests.

## Authors’ contributions

CN drafted the manuscript and was responsible for the overall conception and design of the study and the manuscript. She conducted the literature search and the statistical analyses, and interpreted the study results. FK contributed to the conception and design of the study, participated in the data collection and data management, and revised the manuscript. BR and AW contributed to the conception and design of the study and revised the manuscript. MR contributed to the conception and design of the study and participated in the data collection and data management. All authors read and approved the final manuscript.

## Supplementary Material

Additional file 1 ‘Flowchart Development and Validation of the FHC-Scale’. Click here for file

Additional file 2 ‘Skala zum gesundheitsbezogenen Familienklima (gFk-Skala)’, German version of the FHC-Scale. Click here for file

## References

[B1] Bandura A Social Foundations of Thought and Action: A Social Cognitive Theory 1986 Englewood Cliffs, NJ: Prentice-Hall

[B2] Horn TS Horn JL Tenebaum G, Eklund RC Family Influences on children’s Sport and Physical Activity Participation, Behavior, and Psychosocial Responses Handbook of Sport Psychology 2007 Third Hoboken, New Jersey: John Wiley & Sons 685 711

[B3] Cox MJ Paley B Families as systems Annu Rev Psychol 1997 48 243 267 10.1146/annurev.psych.48.1.243 9046561

[B4] Cox MJ Paley B Understanding families as systems Curr Dir Psychol Sci 2003 12 193 196 10.1111/1467-8721.01259

[B5] Johnson L van Jaarsveld CHM Wardle J Individual and family environment correlates differ for consumption of core and non-core foods in children Br J Nutr 2011 105 950 959 10.1017/S0007114510004484 21110911

[B6] Taylor WC Baranowski T Sallis JF Dishman RK Family Determinants of Childhood Physical Activity: A Social Cognitive Model Advances in Exercise Adherence 1994 Champaign IL: Human Kinetics 319 342

[B7] Ornelas I Perreira KM Ayala GX Parental influences on adolescent physical activity: a longitudinal study Int J Behav Nutr Phys Act 2007 4 3 10.1186/1479-5868-4-3 17274822 PMC1805507

[B8] Vögele C Schwarzer R Ernährung, Körpergewicht und Gewichtsregulation Gesundheitspsychologie. Enzyklopädie der Psychologie, C/X/1 2005 Göttingen: Hogrefe 425 445

[B9] Pearson N Biddle SJH Gorely T Family correlates of fruit and vegetable consumption in children and adolescents: a systematic review Public Health Nutr 2008 12 267 283 18559129 10.1017/S1368980008002589

[B10] Pearson N Biddle SJH Gorely T Family correlates of breakfast consumption among children and adolescents. A systematic review Appetite 2009 52 1 7 10.1016/j.appet.2008.08.006 18789364

[B11] Pugliese J Tinsley B Parental socialization of child and adolescent physical activity: a meta-analysis J Fam Psychol 2007 21 331 343 17874918 10.1037/0893-3200.21.3.331

[B12] Sallis JF Prochaska JJ Taylor WC A review of correlates of physical activity of children and adolescents Med Sci Sports Exerc 2000 32 963 975 10795788 10.1097/00005768-200005000-00014

[B13] McGuire MT Hannan PJ Neumark-Sztainer D Falkner Crossrow NH Story M Parental correlates of physical activity in a racially/ethnically diverse adolescent sample J Adolesc Health 2002 30 253 261 10.1016/S1054-139X(01)00392-5 11927237

[B14] Bradley RH McRitchie S Houts RM Nader P O’Brien M Parenting and the decline of physical activity from age 9 to 15 Int J Behav Nutr Phys Act 2011 8 33 10.1186/1479-5868-8-33 21492482 PMC3095990

[B15] Gustafson SL Rhodes RE Parental correlates of physical activity in children and early adolescents Sports Med 2006 36 79 97 10.2165/00007256-200636010-00006 16445312

[B16] Van der Horst K Oenema A Ferreira I Wendel-Vos W Giskes K van Lenthe F Brug J A systematic review of environmental correlates of obesity-related dietary behaviors in youth Health Educ Res 2007 22 203 226 16861362 10.1093/her/cyl069

[B17] Van der Horst K Paw MJCA Twisk JW Van Mechelen W A brief review on correlates of physical activity and sedentariness in youth Med Sci Sports Exerc 2007 39 1241 1250 10.1249/mss.0b013e318059bf35 17762356

[B18] Ferreira I van der Horst K Wendel-Vos W Kremers S van Lenthe FJ Brug J Environmental correlates of physical activity in youth – a review and update Obesity Rev 2007 8 129 154 10.1111/j.1467-789X.2006.00264.x 17300279

[B19] Tinsley BJ How children learn to be healthy 2003 Cambridge: University Press

[B20] Lohaus A Vierhaus M Ball J Parenting styles and health-related behavior in childhood and early adolescence: results of a longitudinal study J Early Adol 2009 29 449 475

[B21] Schmitz KH Lytle LA Phillips GA Murray DM Birnbaum AS Kubik MY Psychosocial correlates of physical activity and sedentary leisure habits in young adolescents: the teens eating for energy and nutrition at school study Prev Med 2002 34 266 278 10.1006/pmed.2001.0982 11817924

[B22] Resnick MD Bearman PS Blum RW Bauman KE Harris KM Jones J Tabor J Beuhring T Sieving RE Shew M Ireland M Bearinger LH Udry JR Protecting adolescents from harm. Findings from the national longitudinal study on adolescent health J Am Med Assoc 1997 278 823 832 10.1001/jama.1997.03550100049038 9293990

[B23] Kim MJ McIntosh WA Anding J Kubena KS Reed DB Moon GS Perceived parenting behaviours predict young adoelsecents’ nutritional intake and body fatness Mat Child Nutri 2008 4 287 303 10.1111/j.1740-8709.2008.00142.x PMC6860641 18811793

[B24] Kremers SP Brug J de Vries H Engels RC Parenting style and adolescent fruit consumption Appetite 2003 41 43 50 10.1016/S0195-6663(03)00038-2 12880620

[B25] Brustad RJ Who will go out and play? Parental and psychological influences on children’s attraction to physical activity Pediatr Exerc Sci 1993 5 210 223

[B26] Fredricks JA Eccles JS Family socialization, gender, and sport motivation and involvement J Sport Exerc Psychol 2005 27 3 31

[B27] Schunk DH Meece JL Pajares F, Urdan T Self-Efficacy Development in Adolescences Self-Efficacy Beliefs of Adolescents 2005 Greenwich: Information Age Publishing 71 96

[B28] Baranowski T Gochman DS Families and Health Actions Handbook of Health Behavior Research 1: Personal and Social Determinants 1997 New York: Plenum Press 197 206

[B29] Ekvall G Organizational climate for creativity European J Work Organ Psychol 1996 5 105 123 10.1080/13594329608414845

[B30] von Rosenstiel L Haugenbrauck U, Kock K, Kutzner E, Muesmann G Betriebsklima und Leistung – Eine Wissenschaftliche Standortbestimmung Handbuch Betriebsklima 2003 München: Rainer Hampp Verlag 23 38

[B31] Patterson MG West MA Shackleton VJ Dawson JF Lawthom R Maitlis S Robinson DL Wallace AM Validating the organizational climate measure: links to managerial practices, productivity and innovation J Organ Behav 2005 26 379 408 10.1002/job.312

[B32] Parker CP Baltes BB Young SA Huff JW Altmann RA LaCost HA Roberts JE Relationships between psychological climate perceptions and work outcomes: a meta-analytic review J Organ Behav 2003 24 389 416 10.1002/job.198

[B33] Carr JZ Schmidt AM Ford JK DeShon RP Climate perceptions matter: a meta-analytic path analysis relating molar climate, cognitive and affective states, and individual level work outcomes J Appl Psychol 2003 88 605 619 12940402 10.1037/0021-9010.88.4.605

[B34] Anderson CS The search for school climate: a review of the research Rev Educ Res 1982 52 368 420 10.3102/00346543052003368

[B35] Moos RH The Social Climate Scales: An Overview 1974 Palo Alto CA: Consulting Psychologists Press

[B36] Guldenmund FW The nature of safety culture: a review of theory and research Saf Sci 2000 34 215 257 10.1016/S0925-7535(00)00014-X

[B37] Ribisl KM Reischl TM Measuring the climate for health at organizations: development of the worksite health climate scales J Occup Environ Med 1993 35 812 824 10.1097/00043764-199308000-00019 8229333

[B38] Morrison V Bennett P An Introduction to Health Psychology 2009 2 Harlow: Pearson Education

[B39] Summers JA Poston DJ Turnbull AP Marquis J Hoffman L Mannan H Wang M Conceptualizing and measuring family quality of life J Intellect Disabil Res 2005 49 777 783 10.1111/j.1365-2788.2005.00751.x 16162127

[B40] Roth M Entwicklung und Überprüfung einer Kurzform der Familienklimaskalen für Jugendliche (K-FKS-J) Zeitschrift für Differentielle und Diagnostische Psychologie 2002 23 225 234 10.1024//0170-1789.23.2.225

[B41] Moos RH Moos B Family Environment Scale Manual 1981 Palo Alto CA: Consulting Psychologists Press

[B42] Boyd CP Gullone E Needleman GL Burt T The family environment scale: reliability and normative data for an adolescent sample Fam Process 1997 36 369 373 10.1111/j.1545-5300.1997.00369.x 9543658

[B43] Olson DH Portner J Lavee Y FACES III – Family Adaptability and Cohesion Evaluation Scales 1985 St. Paul MN: University of Minnesota

[B44] Schneewind KA Cierpka M Die Familienklimaskalen (FKS) Familiendiagnostik 1987 Berlin: Springer 232 255

[B45] Sonnentag S Pundt A Organizational Health Climate: Construct Validation Studies on Organizational Climates Featuring Healthy Eating, Physical Activity, and Broader Health Behaviors Oral presentation presented at the 10th Industrial and Organizational Psychology (IOP) Conference 2013 Perth, Australia

[B46] Worthington RL Whittaker TA Scale development research: a content analysis and recommendations for best practice Couns Psychol 2006 34 806 838 10.1177/0011000006288127

[B47] Russell DW In search of underlying dimensions: the use (and abuse) of factor analysis in personality and social psychology Personal Soc Psychol Bull 2002 28 1629 1646 10.1177/014616702237645

[B48] Reise SP Waller NG Comrey AL Factor analysis and scale revision Psychol Assess 2000 12 287 297 11021152 10.1037//1040-3590.12.3.287

[B49] Schermelleh-Engel K Moosbrugger H Müller H Evaluating the fit of structural equation models: testing of significance and descriptive goodness-of-fit measures Methods Psychol Res Online 2003 8 23 74

[B50] Chen FF Sensitivity of fit indexes to lack of measurement invariance Struct Equ Model 2007 14 464 504 10.1080/10705510701301834

[B51] Little RJA A test of missing completely at random for multivariate data with missing values J Am Stat Assoc 1988 83 1198 1202 10.1080/01621459.1988.10478722

[B52] Curran PJ West SG Finch JF The robustness of test statistics to nonnormality and specification error in confirmatory factor analysis Psychol Methods 1996 1 16 29

[B53] Vandenberg RJ Lance CE A review and synthesis of the measurement invariance literature: suggestions, practices, and recommendations for organizational research Organ Res Methods 2000 3 4 70 10.1177/109442810031002

[B54] MacCallum RC Austin JT Applications of structural equation modeling in psychological research Annual Rev Psychol 2000 51 201 226 10.1146/annurev.psych.51.1.201 10751970

[B55] Markland D Tobin V A modification to the behavioural regulation exercise questionnaire to include an assessment of amotivation J Sport Exerc Psychol 2004 26 191 196

[B56] Pelletier LG Dion SC Slovinec-D’Angelo M Reid R Why do you regulate what you eat? Relationships between forms of regulation, eating behaviors, sustained dietary behavior change, and psychological adjustment Motiv Emot 2004 28 245 277

[B57] Byrne BM Structural Equation Modeling with AMOS 2010 2 New York: Routledge, Taylor & Francis Group

[B58] Reis HT Collins WA Berscheid E The relationship context of human behavior and development Psychol Bull 2000 126 844 872 11107879 10.1037/0033-2909.126.6.844

[B59] Sallis JF Nader PR Gochman DS Family Determinants of Health Behaviors Health Behavior 1988 New York: Plenum Press 107 124

[B60] Bagozzi RP Heatherton TF A general approach to representing multifaceted personality constructs: application to state self‒esteem Struc Eq Model Multidisc J 1994 1 35 67 10.1080/10705519409539961

[B61] Gribbons BC Hocevar D Levels of aggregation in higher level confirmatory factor analysis: application for academic self‒concept Struc Eq Model Multidisc J 1998 5 377 390 10.1080/10705519809540113

[B62] Lawman HG Wilson DK A review of family and environmental correlates of health behaviors in high-risk youth Obesity 2012 20 1142 1157 10.1038/oby.2011.376 22282044 PMC3360830

[B63] Golan M Weizman A Reliability and validity of the family eating and activity health habits questionnaire Eur J Clin Nutr 1998 52 771 777 10.1038/sj.ejcn.1600647 9805227

[B64] Moreno JP Kelley ML Landry DN Paasch V Terlecki MA Johnston CA Foreyt JP Development and validation of the family health behavior scale Int J Pediatr Obes 2011 6 480 486 10.3109/17477166.2011.575148 21615229

[B65] Repetti RL Taylor SE Seeman TE Risky families: family social environments and the mental and physical health of offspring Psychol Bull 2002 128 330 366 11931522

[B66] Gauze C Bukowski WM Aquan-Assee J Sippola LK Interactions between family environment and friendship and associations with self-perceived well-being during early adolescence Child Dev 1996 67 2201 2216 10.2307/1131618 9022238

[B67] Kurdek LA Fine MA Sinclair RJ School adjustment in sixth graders: parenting transitions, family climate, and peer norm effects Child Dev 1995 66 430 445 10.2307/1131588 7750375

[B68] Franko DL Thompson D Bauserman R Affenito SG Striegel-Moore RH What’s love got to do with it? Family cohesion and healthy eating behaviors in adolescent girls Int J Eating Disord 2008 41 360 367 10.1002/eat.20517 PMC11624907 18318040

[B69] DiMatteo MR Social support and patient adherence to medical treatment: a meta-analysis Health Psychol 2004 23 207 218 15008666 10.1037/0278-6133.23.2.207

[B70] Kraemer HC Measelle JR Ablow JC Essex MJ Boyce WT Kupfer DJ A new approach to integrating data from multiple informants in psychiatric assessment and research: mixing and matching contexts and perspectives Am J Psychiatr 2003 160 1566 1577 10.1176/appi.ajp.160.9.1566 12944328

[B71] Sting S Ecarius J Gesundheit Handbuch Familie 2007 Wiesbaden: VS Verlag für Sozialwissenschaften 480 499

[B72] Campbell TL Kröger F, Hendrischke A, McDaniel S Familie und Gesundheit. Zum Stand der Forschung Familie, System und Gesundheit. Systemische Konzepte für ein Soziales Gesundheitswesen 2000 Heidelberg: Karl Auer Verlag 225 241

[B73] Roest AMC Dubas JS Gerris JRM Value transmission between fathers, mothers, and adolescent and emerging adult children: the role of family climate J Fam Psychol 2009 23 146 155 19364209 10.1037/a0015075

[B74] Schnabel PE Familie und Gesundheit. Bedingungen, Möglichkeiten und Konzepte der Gesundheitsförderung 2001 Weihnheim: Juventa

[B75] Umberson D Gender, marital status and the social control of health behavior Soc Sci Med 1992 34 907 917 10.1016/0277-9536(92)90259-S 1604380

[B76] Trost SG Owen N Bauman AE Sallis JF Brown W Correlates of adults’ participation in physical activity: a review and update Med Sci Sports Exerc 2002 34 1996 2001 10.1097/00005768-200212000-00020 12471307

[B77] Wendel-Vos W Droomers M Kremers S Brug J van Lenthe F Potential environmental determinants of physical activity in adults: a systematic review Obesity Rev 2007 8 425 440 10.1111/j.1467-789X.2007.00370.x 17716300

[B78] Berge JM Wickel K Doherty WJ The individual and combined influence of the ‘quality’ and ‘quantity’ of family meals on adult body mass index Families Syst Health 2012 30 344 351 10.1037/a0030660 PMC3607495 23148980

[B79] Statistisches Bundesamt Zahlen und Fakten https://www.destatis.de/DE/ZahlenFakten/GesellschaftStaat/BildungForschungKultur/Bildungsstand/Tabellen/Bildungsabschluss.html

