# Supplementary figures and images for: Family health climate scale (FHC-scale): development and validation
Source: Int J Behav Nutr Phys Act. 2014 Mar 5;11:30. doi: 10.1186/1479-5868-11-30 (PMC4015295; doi:10.1186/1479-5868-11-30)

Additional File 1 – Flowchart  
Development and Validation of  
the FHC-Scale

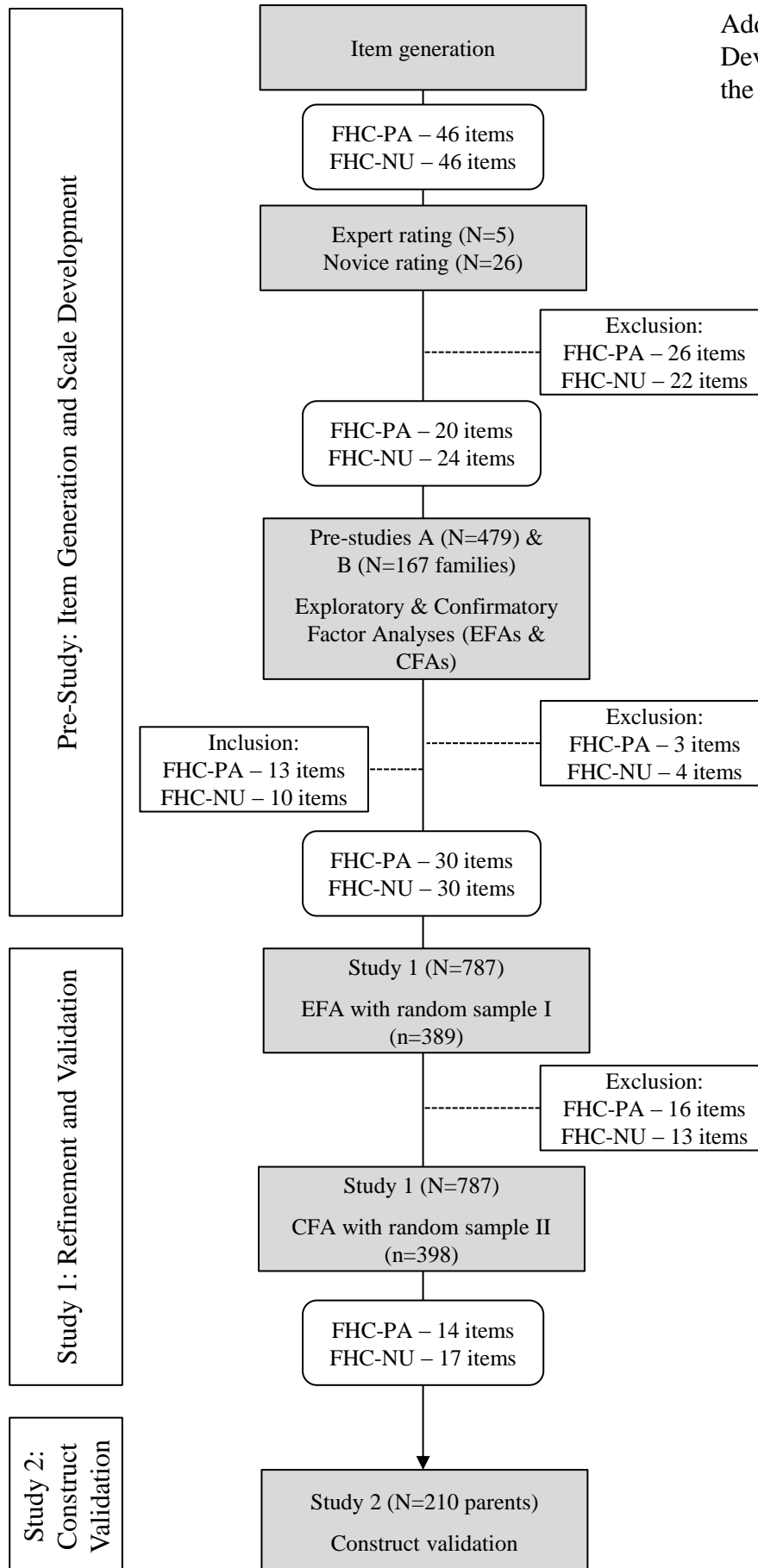

Supplement: Additional file 1 — ‘Flowchart Development and Validation of the FHC-Scale’. [file 1479-5868-11-30-S1.pdf]
