# Supplementary material for: Family health climate scale (FHC-scale): development and validation
Source: Int J Behav Nutr Phys Act. 2014 Mar 5;11:30. doi: 10.1186/1479-5868-11-30 (PMC4015295; doi:10.1186/1479-5868-11-30)
Supplement: Additional file 2 — ‘Skala zum gesundheitsbezogenen Familienklima (gFk-Skala)’, German version of the FHC-Scale. [file 1479-5868-11-30-S2.pdf]

Additional File 2

Skala zum gesundheitsbezogenen Familienklima (gFk-Skala)

In unserer Familie…

Körperliche Aktivität (gFk-A)	Ernährung (gFk-E)
**Stellenwert**	**Stellenwert**
[1] …legen wir großen Wert darauf, uns im Alltag viel zu bewegen.	[1] …spielt eine gesunde Ernährung eine große Rolle.
[2] …ist es ganz normal, dass man regelmäßig körperlich aktiv ist.	[2] …achten wir automatisch darauf, dass wir uns gesund ernähren.
[3] …ist es selbstverständlich, regelmäßig Sport zu treiben und sich körperlich zu betätigen.	[3] …ernähren wir uns gewohnheitsmäßig gesund.
[4] …gehört es einfach dazu, dass wir uns in unserer Freizeit körperlich betätigen.	[4] …ist es ganz normal, dass wir zu gesunden Lebensmitteln greifen.
[5] …sind wir einer Meinung, dass sportliche Aktivitäten zum Alltag dazu gehören.	
**Zusammenhalt**	**Zusammenhalt**
[1] …mögen wir das Miteinander bei gemeinsamen körperlichen Aktivitäten (z.B. bei Radtouren, Wanderungen).	[1] …schätzen wir das Miteinander beim Essen.
[2] …sind wir gerne gemeinsam sportlich aktiv.	[2] …nimmt jeder gerne an gemeinsamen Mahlzeiten teil.
[3] …haben wir Spaß bei gemeinsamen körperlichen Aktivitäten (z.B. bei Radtouren, Wanderungen).	[3] …gehören gemeinsame Mahlzeiten zu unserem Familienalltag dazu.
[4] …finden wir es schön, gemeinsam sportlich aktiv zu sein.	[4] …schmeckt es uns am besten, wenn wir gemeinsam am Tisch sitzen.
[5] …verbringen wir gerne Zeit miteinander bei gemeinsamen sportlichen Aktivitäten.	[5] …versuchen wir so oft wie möglich gemeinsam zu essen.
**Information**	**Kommunikation**
[1] …schauen wir im Fernsehen Sendungen zum Thema „Bewegung und Sport“.	[1] …interessieren wir uns für Beiträge (z.B. in Zeitschriften) zum Thema "gesunde Ernährung".
[2] …suchen wir gezielt nach aktuellen Erkenntnissen zum Thema „Sport und Bewegung“, um auf dem neuesten Stand zu bleiben.	[2] …erinnern wir einander, auf eine gesunde Ernährung zu achten.
[3] …sammeln wir Informationen (z.B. im Internet etc.) zum Thema „Sport und Bewegung“.	[3] …reden wir darüber, welche Lebensmittel gesund sind.
[4] …lesen wir Zeitungsartikel, Zeitschriftenbeiträge o. ä., in denen es um Fitness, Bewegung und Sport geht.	[4] …unterstützen wir einander dabei, auf ungesunde Dinge zu verzichten.
	[5] …sprechen wir darüber, wie wir uns gesund ernähren können.
	**Einigkeit**
	[1] …gibt es selten Auseinandersetzungen in Bezug auf Essens- und Ernährungsangelegenheiten.
	[2] …sind wir uns in Ernährungsfragen einig.
	[3] …sind wir meist einer Meinung, wenn es um unser Essen und die Auswahl der Lebensmittel geht.

Rating-Skala: (0) trifft nicht zu; (1) trifft eher nicht zu; (2) trifft eher zu; (3) trifft zu
